# Suboptimal glycaemic and blood pressure control and screening for diabetic complications in adult ambulatory diabetic patients in Uganda: a retrospective study from a developing country

**DOI:** 10.1186/2251-6581-13-40

**Published:** 2014-03-04

**Authors:** Davis Kibirige, David Atuhe, Robert Sebunya, Raymond Mwebaze

**Affiliations:** 1 Department of Medicine, Uganda Martyrs Hospital Lubaga, P.O. Box 7146, Kampala, Uganda; 2 Department of Medicine, St. Raphael of St. Francis hospital Nsambya, Kampala, Uganda; 3 Mother Kevin Postgraduate Medical School, Uganda Martyrs University Nkozi, Nkozi, Uganda; 4 Diabetes and endocrine clinic, St. Raphael of St. Francis hospital Nsambya, Kampala, Uganda

**Keywords:** Glycaemic and blood pressure control, Screening, Diabetic complications and Uganda

## Abstract

**Background:**

Currently, Sub Saharan Africa is faced with a substantial burden from diabetes mellitus. In most of the African countries, screening for diabetes related complications and control of blood pressure and glycaemic levels is often suboptimal.

The study aimed at assessing the extent of optimal glycaemic and blood pressure control and the frequency of screening for diabetic complications in adult ambulatory Ugandan diabetic patients.

**Methods:**

This was a retrospective study of 250 medical records of adult diabetic patients attending the outpatient diabetic clinic at St. Raphael of St. Francis hospital Nsambya in Kampala, Uganda.

**Results:**

The mean age of the patients was 51.6 ± 9.2 years with the majority being females (155, 62%). Using fasting blood glucose levels assessed in all the patients, optimal glycemic control of <7.2 mmol/l was noted in 42.8% of the patients. Glycated haemoglobin was performed at least once in the last year in 24 (9.6%) patients , of which 5 (20.8%) of these attained optimal control of <7%. Optimal blood pressure (BP) control defined as BP ≤140/80 mmHg was noted in 56% of the patients. Hypertension and diabetic neuropathy were the most screened for diabetic complications in 100% and 47.2% of the patients respectively and were also the most prevalent diabetic complications (76.4% and 31.2% respectively).

**Conclusions:**

This study demonstrates that glycemic and blood pressure control and screening for diabetic complications among the adult ambulatory diabetic patients in this urban diabetic clinic is suboptimal. This substantiates development and implementation local guidelines to improve diabetes care.

## Introduction

There is an estimated exponential increase in the proportion of people living with diabetes mellitus (DM) in the next two decades with the greatest disease burden to be documented in low and middle income countries. According to the International Diabetes Federation (IDF) in 2011, an estimated 366 million people have DM. This figure is projected to rise to 552 million by 2030 [1].

People living with DM in Sub Saharan Africa (SSA) are faced with a disproportionate risk of lethal and disabling diabetic complications such as the diabetic foot, nephropathy, retinopathy, coronary artery disease and cerebrovascular disease. These lead to significant mortality among patients with DM [2] and also have a colossal strain on the existing meager health resources [3].

There is a great need to improve diabetes care among patients with DM in SSA in order to minimise the development of macro and microvascular diabetic complications. Studies on the quality of diabetes care from Africa have documented suboptimal screening for diabetic complications and control of glycemic, lipid and blood pressure levels among diabetic patients [4-8].

In Uganda, a low income developing country, there is also paucity of published information on the extent of glycaemic and blood pressure control, frequency of screening for diabetes complications and magnitude of diabetic complications despite the documented growing prevalence of DM in the country.

We therefore performed a retrospective study on 250 selected medical records of adult diabetic patients at St. Raphael of St. Francis hospital Nsambya in Kampala, Uganda. Its aim was to assess the extent of optimal glycaemic and blood pressure control and the frequency of screening for diabetic complications in reference to the recently published 2013 American Diabetes Association (ADA) guidelines of standard of medical care of DM (Table 1) [9]. This information will help propose evidence to help in the formulation of institutional guidelines of diabetes care and to institute standard practices of DM management. This will also directly reduce morbidity and mortality due to DM complications among our adult diabetic patients.

**Table 1:** 2013 American Diabetes Association guidelines of standards of medical care of diabetes mellitus

**A. Glycemic goals**	**B. Blood pressure goals**	**C. Lipid profile goals**	**D. Laboratory evaluation**
1. HbA1c ≤ 7% or a pre-prandial capillary plasma glucose level of 3.9-7.2 mmol/l	1. Blood pressure of systolic ≤140 and diastolic ≤ 80 mmHg	1. LDLC levels of < 100 mg/dl or 2.6 mmol/l in absence of cardiovascular diseases (CVD) or < 70 mg/dl or 1.8 mmol/l in individuals with overt CVD	1. Measurement of the glycated haemoglobin (HbA1c) levels at least once annually. 2. Annual assessment of fasting lipid profile (triglyceride, total cholesterol and high density lipoprotein cholesterol (HDLC) and low density lipoprotein cholesterol (LDLC) levels). 3. Annual assessment of renal function status by measurement of serum creatinine and calculate an estimated glomerular filtration rate.

### Study methods

This study was a descriptive retrospective study of 250 selected medical records of adult diabetic patients who regularly attended the diabetic clinic within a recent period of 1 year at St. Raphael of St. Francis hospital Nsambya in Kampala, Uganda. It was carried out between June and July 2013. The hospital is a private not-for-profit hospital located in Kampala, the capital city of Uganda and serves a predominantly middle and high income urban population of about 1 million people.

The diabetes clinic operates once a week and is managed by 2 diabetic nurses, intern doctors, internal medicine residents and internists. An average of 60 patients is seen on each clinic day. Prior to being reviewed by the doctors, all patients receive diabetic health education offered by the trained diabetic nurses.

The charts of regularly attending patients were reviewed for a recent documentation of the parameters to assess the extent of glycemic and blood pressure control. Fasting blood sugar and glycated haemoglobin (HbA1c) levels as measures of glycaemic control were assessed using a point of care Accu-Check Active glucometer by Roche diagnostics and Roche immunoassay at the hospital’s central laboratory respectively. Blood pressure measurement of the patients was performed using a mercury sphygmomanometer after a 15 min rest.

The frequency of screening for diabetic complications within a period of 1 year was also recorded. The complications included hypertension, diabetic retinopathy, diabetic neuropathy, diabetic nephropathy, diabetic foot complications; cardiac complications-ischemic heart disease, dyslipidemia, peripheral vascular disease and stroke. Newly diagnosed and pre-existing diabetes related complications on screening were also documented. A comprehensive drug review was also performed.

A diagnosis of diabetic neuropathy was made using mono filament testing or by assessing for the presence of neuropathic symptoms like burning, pricking sensation or numbness in history. Diabetic retinopathy and nephropathy among the patients were screened using dilated eye examination using fundoscopy and measurement of serum creatinine and macroalbuminuria respectively. Diabetic foot complications and peripheral arterial disease were assessed using clinical inspection for wounds, ulcers, changes in skin colour and temperature, loss of toe hair and direct palpation of feet pulses. Ischemic heart disease and stroke was assessed using history of suggestive symptoms, medical records and electrocardiography if available.

The criteria used to diagnose diabetes in all these patients prior to enrolment into the diabetic clinic was either a fasting random blood sugar ≥ 126 mg/dl, a random blood sugar level ≥ 200 mg/dl or HbA1c ≥ 6.5% in the presence of symptoms of diabetes like polyuria and polydipsia. Regularly attending patients were defined as those who had a minimum of 6 documented clinical reviews at the clinic in the period of 1 year. We selected 250 medical charts of patients who fulfilled that study definition whose information was extracted and analysed.

### Study statistics

Data was entered into an access data base, FoxPro for windows (Version 2.6; Microsoft) and Stata software, version 12.1 was used for all statistical analysis. Patient characteristics were reported as frequencies and percentage for most variables.

### Ethical considerations

Approval to carry out this study was sought and obtained from the Research and Ethics Committee of St. Raphael of St. Francis hospital Nsambya, Uganda.

## Results

### Baseline and clinical characteristics of the patients

Majority of the patients were females (155[62%]). The mean age was 51.6 ± 9.2 years with the youngest being 21 years and the oldest 89 years. One hundred forty (56%) and 235 (94%) patients were employed and had attained some level of formal education respectively. Only 3 (1.2%) and 22 (8.8%) patients were documented to be current and former smokers respectively. Family history of DM was documented in only 93 (37.2%) patients. Majority of the patients had type 2 DM (243, 97%) and a normal body mass index (BMI)- (130[52%]). The mean BMI of the patients was 24.5 ± 3.9 kg/m^2^ (Table 2).

**Table 2:** Baseline and clinical characteristics of the study participants (n = 250)

**Characteristic**	**Frequency**	**Percentage**
Age, years		
Mean ± SD: 51.6 ± 9.2		
18-64	158	62.2
>65	92	36.8
Gender		
Female	155	62
Occupation		
Employed	140	56
Education status		
Educated	235	94
Smoking status		
Former smokers	22	8.8
Current smokers	3	1.2
Family history of diabetes		
Yes	93	37.5
Body mass index in kg/m^2^		
Mean ± SD 24.5 ± 3.9		
17-24.9	130	52
25-29.9	95	38
>30	25	10
Category of diabetes		
Type 2 DM		97

*DM-Diabetes Mellitus.

### Frequency of screening and attainment of the optimal glycaemic and blood pressure goals

#### Frequency of screening and adequacy of glycemic control

Fasting blood glucose (FBG) levels were assessed in all the patients during the most recent clinical review. Optimal glycemic control as defined by a FBG ≤7.2 mmol/l was documented in 107 (42.8%) participants. The mean FBG was 9.9 ± 5.3 mmol/l.

Glycated haemoglobin (HbA1c) as a measure of assessing glycemic control was performed at least once in the most recent year in 24 (9.6%) patients. Only 5 (20.8%) of these had optimal glycemic control as defined by HbA1c of <7%. The mean HbA1c of the patients was 9.9 ± 2.9%.

Apart from 2 (0.8%) patients who were on conservative management using diet and exercise, the rest received pharmacological blood lowering treatment. Majority were receiving oral hypoglycemic drugs (198, 79.6%) with the combination of metformin and a sulphonylurea being the most frequently used (141, 71.2%). Metformin monotherapy was used in 57 (22.8%) patients. A combination of metformin and pre-mixed insulin (Mixtard®) was used in 42 (16.8%) patients while only 8 (3.2%) were on insulin (Mixtard®) monotherapy. Long acting basal insulin regimens, incretin based therapies and α glucosidase inhibitors were not used in any patient.

#### Frequency of screening and adequacy of blood pressure control

All patients received blood pressure measurements at each clinical visit. Optimal blood pressure control as defined as a blood pressure of ≤140/80 was noted in 140 (56%) patients. Fifty two (20.8%) patients attained both optimal glycaemic and blood pressure control. The mean systolic and diastolic blood pressures were 137.1 (SD 22.1) and 80.7 (SD 12.4) respectively.

Majority of the patients (196[78.4%]) were receiving at least an anti-hypertensive drug. An angiotensin converting enzyme inhibitor (ACEI) or angiotensin receptor blocker (ARB) and calcium channel blocker (CCB) combination and an ACEI or ARB monotherapy were the most frequently used drug regimens in 79 (40.3%) and 48 (24.5%) respectively. Only 6 (2.4%) patients received an anti hypertensive regimen without an ACEI or ARB (Table 3).

**Table 3:** Blood glucose and pressure lowering drugs used by the study participants (n = 250)

	**Frequency**	**Percentage**
Blood glucose lowering drugs		
OHAs alone		
Yes	198	79.6
Type of OHA used		
Metformin and a SU	141	71.2
Metformin only	57	28.8
Insulin monotherapy		
Yes	8	3.2
Pre-mixed insulin and metformin		
Yes	42	16.8
Blood pressure lowering drugs		
On therapy		
Yes	196	78.4
Class of anti hypertensive used.		
ACEI/ARB monotherapy	48	19.2
ACEI/ARB and CCB	79	31.6
ACEI/ARB and beta blocker	2	0.8
ACEI/ARB and thiazide diuretic	27	10.8
ACEI/ARB, thiazide diuretic and CCB	29	11.6
ACEI/ARB, thiazide diuretic, CCB and beta blocker	1	0.4
ACEI/ARB, CCB and beta blocker	5	2
Other anti hypertensives without ACEI/ARBS	6	2.4

OHAs- oral hypoglycemic agents SU- sulphonylurea ACEI-angiotensin converting enzyme inhibitor ARB- angiotensin II receptor blocker CCB-calcium channel blocker.

The use of low dose cardiac aspirin for either primary or secondary prophylaxis against cardiovascular diseases was documented in 95 (38%) patients. Low dose cardiac aspirin, a statin and anti hypertensives were simultaneously used in only 36 (14.4%) patients.

#### Frequency of screening for diabetic complications

Hypertension was the most frequently screened for diabetes related complication, performed in all the patients. Diabetic neuropathy was the second most screened complication in 118 (47.2%) patients.

Only 35 (14%) patients had their lipid profiles assessed for dyslipidemia at least once in a period of 1 year. Results for only 15 (42.9%) of these screened patients were available in the medical records. Optimal lipid control as defined as low density lipo-protein cholesterol (LDLC) concentrations ≤100 mg/dl was noted in only 3 (20%) patients. Fifty one (20.4%) patients were on statin therapy as a lipid lowering therapy. No patient was on a fibrate or a fibrate and statin combination.

Thirty one (12.4%) patients were screened for diabetic nephropathy at least once in 1 year and measurement of the serum creatinine levels was the most frequently used mode of assessment in 80.6% of the patients. No patient was assessed for diabetic nephropathy using microalbuminuria measurement. Screening for diabetic retinopathy (using fundoscopic examination), peripheral vascular disease (basing on clinical examination for skin colour and hair changes of the feet and character of the feet pulses), cardiac complications (using medical records, an electrocardiography or/and echocardiography) and feet examinations for diabetic foot ulcers were performed at least once in 1 year in 14%, 18%, 5.6% and 21.2% of the patients respectively.

#### Pre-existing and newly diagnosed diabetic complications on screening among the patients

Hypertension both newly diagnosed and pre-existing (controlled and uncontrolled) was the most documented complication in 191 (76.4%) patients. Diabetic neuropathy, retinopathy and nephropathy were present in 31.2%, 9.6% and 2% of the patients respectively.

Among those screened for diabetic retinopathy, cataracts and non proliferative retinopathy were present in 16 (6.4%) and 14 (5.6%) patients respectively. Only 1 (0.4%) patient had glaucoma. Diabetic foot complications were reported in 8 (3.2%) patients with majority of these having diabetic ulcers-Wagner classification I-III (6, 75%).

Diabetic macrovascular complications were very infrequent among the patients. Peripheral vascular disease, ischemic heart disease and stroke were reported in 2%, 1.2% and 0.4% of the patients. The frequency of all the complications is illustrated in Table 4.

**Table 4:** Newly diagnosed and pre-existing diabetic complications (n = 250)

**Complication**	**Frequency (n)**	**Percentage, %**
**Hypertension**		
Yes	191	76.4
Diabetic neuropathy		
Yes	78	31.2
Myocardial Infarction		
Yes	3	1.2
Diabetic retinopathy		
Yes	24	9.6
Type of retinopathy		
Cataracts	16	6.4
Non proliferative type	7	2.8
Glaucoma	1	0.4
Stroke or TIA*		
Yes	1	0.4
Diabetic foot		
Yes	8	3.2
Diabetic nephropathy		
Yes	2	0.8
Peripheral vascular disease		
Yes	5	2

*TIA- transient ischemic attack.

## Discussion

In this descriptive retrospective study, we explored the extent of optimal glycaemic and blood pressure control and frequency of screening for diabetic complications in adult ambulatory diabetic patients attending a diabetic outpatient clinic at an urban hospital in Uganda. This information evidently demonstrates that according to the 2013 ADA guidelines of diabetes care, glycemic and blood pressure control and screening for diabetic complications is suboptimal in this clinical setting. Attainment of the recommended glycemic and blood pressure targets is often not easy in most settings even in developed countries with adequate health care resources [10].

### Glycaemic control

In our study population, optimal glycemic control using HbA1c and FBG levels was noted in 20.8% and 42.8% of the participants respectively. The probable reasons to explain the poor glycemic control among this study population are multifactorial. Financial constraints are a key factor since all the patients have to buy their drugs. The prices of some of these drugs are prohibitive to some patients. Poor adherence to therapy could also explain the poor glycemic control. Poor prescription patterns of health personnel could also be another reason. Insulin prescription despite poor glycaemic control is relatively infrequent among adult diabetic patients demonstrating the aspect of “clinical inertia” among majority of the health personnel. In our study, only 3.2% and 16.8% of the patients received insulin either as monotherapy or in combination with metformin respectively.

Suboptimal glycemic control has also been noted in similar African studies performed in past years. In studies performed to assess the quality of diabetes care in specialised diabetes centres in Libya [11] and Ethiopia [7], optimal glycemic control as assessed using FBG levels similar to what was used in all of our study participants was 9.2% and 26.9% respectively. The extent of optimal glycemic control in these studies is inferior to what we noted in our study population. HbA1c testing in this urban hospital is available though expensive to some of the patients. A low inclination to performing HbA1c measurement among the diabetic patients by the health personnel could also probably explain why few patients had HbA1c measurement as a measure of assessing glycaemic control.

Other similar studies, though cross sectional in nature reported from South Africa [12] and Nigeria (the Diabcare Nigeria study) [6] documented optimal glycemic control (defined as HbA1c <7%) in 30.4% and 32.4% of their study populations respectively. The largest multicentre descriptive cross sectional study of 2,352 type 2 diabetic patients performed in 6 sub Saharan African countries to date to study the quality of diabetes control and co-existing diabetes related complications (the Diabcare Africa study) reported optimal glycemic control (defined as HbA1c level < 6.5%) in only 29% of the study participants [5].

### Optimal blood pressure control

Optimal blood pressure control was noted in 56% of the patients as per the 2013 ADA guidelines. The proportion of patients who attained optimal blood pressure control is higher than what has been reported in most African studies [5-7]. The probable reason for this variation is the different study definition of optimal control used. The previous ADA guidelines recommended a target blood pressure of ≤130/80 mmHg which was used in all the quoted African studies.

In agreement with the guidelines regarding prevention or retarding progression of diabetic nephropathy, a good proportion of the patients received anti hypertensive therapy with an ACEI/ARB (76%). Hypertension as a co-existing complication was highly prevalent in our study population (76.4%). Majority of the African studies have documented similar findings of high frequency of hypertension among adult diabetics ranging from 44.4% to 65% [5-8].

### Frequency for screening for and prevalence of diabetic complications

With the exception of hypertension, the frequency of screening for diabetic complications in the last 1 year was incredibly low. Less than 50% of the patients were screened for diabetic neuropathy despite its being the second most prevalent diabetic complication in this study population (31.2%). Diabetic retinopathy and nephropathy were screened in less than 15% of the patients.

Only 21.2% of the patients had at least one foot examination and eight (3.2%) patients were noted to have diabetic foot complications with diabetic ulcers being the commonest (6, 75%). The most probable aetiology of the diabetic foot complications in this study population was diabetic neuropathy due to its high prevalence (31.2%). A small fraction of patients (2%) had features of peripheral vascular insufficiency on clinical examination. This finding of low prevalence of diabetic foot complications predominantly of neuropathic type has been replicated in most African studies in adult diabetic populations [5].

Screening for dyslipidemia, a complication that is highly prevalent in DM patients was performed only 35 (14%) study participants, with results documented in only 15 participants. Of these 15 participants, only 3 (42.9%) had optimal LDLC levels of < 100 mg/dl as per the ADA guidelines. Lipid lowering therapy (a statin) was prescribed in 20.4% of the 250 study participants.

Most African studies have reported very low rates of screening for dyslipidemia and use of lipid lowering rates. In the Diabcare Africa study, 45% of the patients had their lipid profile assessed at least once in a year with only 13% on lipid lowering therapy [5]. In the Diabcare Nigeria study, 48% of the patients were screened for dyslipidemia with low high density lipoprotein cholesterol and hypertriglyceridemia being the most prevalent lipid abnormalities. Treatment for hyperlipidemia was reported in only 12.6% of the patients [6].

Similarly, a low frequency of screening for dyslipidemia and use of lipid lowering drugs of 4.9% and 2.7% respectively was reported in one study done in Ethiopia [7]. However, in another study performed in Northern Ethiopia, no cases of hyperlipidemia were found among 105 adult diabetic patients [13]. This universally low rate of screening for hyperlipidemia and prescription of statins among adult diabetics in Africa calls for a need of an urgent intervention in diabetes care. The cost of assessing lipid profile and purchasing statins and the health personnel’s low inclination to prescribe statins could explain these findings.

Screening for macro vascular diabetic complications was also exceedingly low. Peripheral vascular disease and cardiac diseases were screened in 18% and 5.6% of the patients. On screening, macrovascular diabetic complications were noted to be very uncommon in this study population. Peripheral vascular disease, ischemic heart disease and stroke or transient ischemic attack was documented in only 2%, 1.2% and 0.4% of the patients respectively. This remarkably low frequency of screening for and prevalence of macrovascular diabetic complications have also been described in most African studies [5-7,13]. In the Diabcare Africa [5] and Diabcare Nigeria [6] studies, stroke, myocardial infarction or coronary artery by-pass graft or angiography was reported in less than 5% of the patients. No evidence of macrovascular diabetic complications was found in an adult diabetic population in one study performed in North Ethiopia [13].

This low frequency among adult African diabetic patients and sub Saharan African (SSA) population in general could be explained by the genetic influence, low frequency of smoking, lipid abnormalities and screening in clinical practice.

## Conclusion

In this single centre study, glycemic and blood pressure control and screening for diabetic complications among ambulatory adult diabetics was suboptimal. Majority of the patients were hypertensive and had diabetic neuropathy. Macrovascular diabetic complications were very infrequent.

Local diabetes care policies adapted from the ADA guidelines of diabetes management should be urgently developed and implemented. Regular comprehensive diabetic education to patients and diabetic health programs for health care personnel should be encouraged as principal steps in improving health outcomes and quality of diabetes care. Provision of point of care HbA1c and lipid profile testing in most diabetic clinics in the country may also improve on patient monitoring and treatment outcomes.

### Study limitations

Due to the study design, our study is absolutely associated with the similar demerits of retrospective studies. Due to the small sample size and selection bias, these findings cannot be extrapolated to general diabetes care in Uganda.

## Competing interests

The authors declare that they have no competing interest.

## Authors’ contributions

DK, AD, RS and RM- Collectively developed the concept of the study, collected data and participated in writing the manuscript. All authors approved the final draft of the manuscript.
